# The Chemoreceptive Molecular Mechanism Underlying CSP-Mediated Recognition of Seed Elaiosome from *Stemona tuberosa* by Hornets

**DOI:** 10.3390/genes16111265

**Published:** 2025-10-27

**Authors:** Guangyan Long, Yuying Liu, Mengyao Zhu, Kaiyu Liu, Yutao Xiao, Hui Ai

**Affiliations:** 1 College of Plant Science and Technology, Huazhong Agricultural University, Wuhan 430070, China; 2 Agricultural Genomics Institute at Shenzhen, Chinese Academy of Agricultural Sciences, Shenzhen 518000, China; 3 Key Laboratory of Pesticide & Chemical Biology of Ministry of Education, College of Life Sciences, Central China Normal University, Wuhan 430079, China

**Keywords:** hornets, *Vespa velutina auraria*, chemosensory protein, plant seed elaiosome, olfactory recognition

## Abstract

Background/Objectives: As crucial natural predators, hornets contribute to ecosystem function by preying on agricultural and forest pests and facilitating plant pollination. However, the predatory preference of hornets for honeybees poses a significant threat to honeybee pollination and the development of the beekeeping industry. Foraging and pollination behaviors in hornets are largely governed by a sensitive olfactory system, but their olfactory molecular mechanisms remain poorly understood. Methods: VvelCSP1 and VvelCSP4 were successfully expressed in the prokaryotic expression system and purified by Ni-NTA affinity chromatography column. Fluorescence competitive binding assays were employed to evaluate their binding affinities to volatile compounds derived from the seed elaiosome of *Stemona tuberosa* and honeybees. Molecular docking was further performed to analyze key residues and interaction patterns within the binding pockets. Results: Fluorescence competitive binding assays showed that both proteins prefer long-chain alkanes yet exhibit significant substrate selectivity and high ligand specificity. VvelCSP1 specifically binds to hexacosane, while VvelCSP4 specifically recognizes docosane. Molecular docking results demonstrated that the binding process between VvelCSP1, VvelCSP4 and their respective ligands is dominated by hydrophobic interactions. Conclusions: This study provides functional evidence for investigating the olfactory molecular regulation mechanisms underlying hornet-mediated seed dispersal. These findings establish a foundation for potential applications of hornets in plant propagation, biological pest control, crop pollination and ecological balance maintenance in agroforestry systems.

## 1. Introduction

As generalist predators in natural agroforestry ecosystems, hornets have been extensively documented for their roles in pest control and seed dispersal, while also posing significant threats to honeybees and the beekeeping industry [1,2,3,4,5]. These feeding behaviors underscore their ecological significance in both biological pest control and pollination services within agroforestry systems. During foraging, hornets efficiently associate food resources with specific visual, spatial and olfactory cues, enabling precise identification and localization of prey and nutrients [6]. For instance, *Vespula germanica*, *Vespa mandarinia*, *Vespa simillima* and *Vesp analis* rely heavily on olfactory signals to explore novel foraging sites and recruit nestmates while integrating visual and olfactory inputs for final prey targeting and capture near food sources [7]. Kim et al. reported that *Vespula koreensis* foragers initially prefer easily accessible fruit-based diets but later shift toward more challenging targets such as floral nectar and agricultural insects [8]. As a generalized predator, the Asian hornet (*Vespa velutina auraria*) employs a sophisticated sensory system for food localization and recognition. While previous studies have focused on its predatory behavior, geographical distribution and invasive potential [9,10,11], the molecular and sensory mechanisms underlying its olfactory recognition system, particularly in detecting flowering plants, honeybees and crop pests, remain poorly understood.

Chemosensory proteins (CSPs) represent a class of small, soluble ligand-binding proteins crucial for chemoreception. They are characterized by a compact structure stabilized by two disulfide bonds formed from four conserved cysteine residues, creating a framework of interacting helices and loops [12,13,14]. Functional analyses of multiple CSPs in the Chinese honeybee (*Apis cerana*) have provided significant insight; for example, AcerCSP1, AcerCSP2, and AcerCSP4 are abundantly expressed in the antennae of honeybees [15,16]. Immunohistochemical localization indicates that AcerCSP3 is primarily expressed in mechanosensory sensilla on the antennae. Competitive binding assays demonstrate that AcerCSP1-4 proteins exhibit strong binding affinities for various floral volatile compounds, including β-ionone, 3,4-dimethylbenzaldehyde and methyl salicylate, implicating these CSPs in the olfactory recognition of flowering plants [16,17]. Additionally, AcerCSP1 binds strongly to two queen pheromone components and can recognize plants like osmanthus, chrysanthemum and tomato via binding to the plant volatile 3-carene [16]. Although binding characteristics of CSPs in the *Apis mellifera* are less explored, comparative sequence analyses reveal high similarity between *A. cerana* and *A. mellifera* CSPs, suggesting significant functional conservation during the evolution of pollinating bees [16,18]. Beyond olfaction, AmelCSP5 is expressed in the ovaries and may be involved in ovarian development and embryonic cuticle formation, while AmelCSP3 acts as a brood pheromone carrier, specifically binding fatty acids and their ester derivatives that constitute the pheromone blend [19,20]. Emerging evidence indicates that chemosensory proteins (CSPs) are involved in diverse physiological processes in insects, including olfaction and lipid transport, suggesting their potential role in the chemical ecology of hornet foraging and host-seeking behavior.

Previous field observations, biochemical analyses, and behavioral experiments have established that the hornet *V. velutina auraria* mediates long-distance seed dispersal of the medicinal plants *Stemona tuberosa* and *Aquilaria sinensis* [21,22,23]. The volatile semiochemicals released are likely critical signals maintaining the mutualistic relationship between hornets and the seeds, a phenomenon also observed in other hornet species like *Vespula flaviceps*, *V. mandarinia* and *Vespa bicolor* [24]. Moreover, *S. tuberosa* seeds exhibit morphological mimicry of typical hornet prey, and their elaiosomes release volatile compounds similar to those on the honeybee cuticle to attract hornets, thereby facilitating seed dispersal [24]. The present study employs molecular biology techniques, including prokaryotic expression purification and fluorescent competitive binding assays, to functionally characterize the CSPs involved in hornet foraging and seed dispersal behaviors. Our primary objectives are to elucidate the behavioral mechanisms underlying hornet-mediated long-distance seed dispersal of *S. tuberosa* and to decipher the molecular regulatory mechanisms of chemoreception governing hornet predation. This research aims to provide fundamental insights that will support the sustainable utilization and conservation of hornet resources.

## 2. Materials and Methods

### 2.1. Insect Rearing and Tissue Collection

The freshly adult hornets used in this study were provided by our collaborator, Professor Gao Chen, from the Kunming Institute of Botany, Chinese Academy of Sciences. Worker hornets were reared in climate-controlled chambers with controlled environmental conditions (temperature: 26 ± 1 °C; photoperiod: 14 L:10 D; relative humidity: 60% ± 10%) and supplied with diluted honey water. A total of thirty female worker hornets (3–5 days post-emergence) were collected for antenna sampling. The collected antennae were pooled into three biological replicates, each containing antennae from ten hornets.

### 2.2. RNA Extraction and cDNA Synthesis

RNA was extracted from hornet antennae using TRIzol reagent (Invitrogen, Carlsbad, CA, USA) according to the manufacturer’s protocol. RNA quality and concentration were assessed using a NanoDrop 2000 spectrophotometer (NanoDrop, Wilmington, DE, USA, and 1.1% agarose gel electrophoresis. Subsequently, the RNA was reverse-transcribed into cDNA using the EasyScript One-Step gDNA Removal and cDNA Synthesis SuperMix (TransGen, Beijing, China), strictly following the manufacturer’s protocol. The synthesized cDNA was stored at −80 °C.

### 2.3. Sequence Alignment and Motif Analysis

The amino acid sequences of VvelCSP1 (accession no. XM_047500533.1) and VvelCSP4 (accession no. XM_047501689.1) were retrieved from the National Center for Biotechnology Information (NCBI) database. These sequences were then subjected to a series of bioinformatic analyses. Signal peptides of the CSPs were predicted using the SignalP-6.0 online server (https://services.healthtech.dtu.dk/services/SignalP-6.0/, accessed on 24 March 2025). Multiple sequence alignment of the CSP amino acid sequences was performed with DNAMAN software (version 9.0, Lynnon Biosoft, San Ramon, CA, USA), followed by sequence analysis and visualization using GeneDoc software v2.7. The analysis of conserved motifs in the CSP sequences and their homologs was conducted using the MEME suite (meme-suite.org https://meme-suite.org/tools/meme, accessed on 28 September 2025), and the results were visualized and exported as a motif distribution map using TBtools (v2.363) software.

### 2.4. Prokaryotic Expression and Purification of VvelCSPs

The coding sequences of *VvelCSP1* and *VvelCSP4* genes, synthesized by GenScript Biotech (GenScrip, Nanjing, China), were cloned into the pET-32a(+) vector using restriction digestion and T4 DNA ligase with the primers listed in Supplementary Table S1 [25]. The constructed recombinant plasmids were transformed into *Escherichia coli* BL21(DE3) competent cells for protein expression. Positive single colonies, verified by PCR and sequencing, were cultured in LB liquid medium with ampicillin at 37 °C and 220 rpm. Protein expression was induced overnight with IPTG (0.8 mM) at 25 °C, when the OD_600_ reached 0.6–0.8, with optimal conditions determined by small-scale tests. Post-induction cells were centrifuged, and the pellets were resuspended in PBS buffer (pH 7.4). Cells were lysed by ultrasonication, the lysate was centrifuged, and the supernatant was loaded onto a Ni-NTA affinity chromatography column (Vdobiotech, Suzhou, China). The column was equilibrated and washed with buffers containing an imidazole gradient (20–50 mM), and target proteins were eluted using 250–500 mM imidazole buffer. Eluted proteins were dialyzed against PBS buffer at 4 °C for over 13 h, analyzed via 12% SDS-PAGE, and stored at −80 °C. Protein expression and purity were verified using Coomassie Brilliant Blue staining.

### 2.5. Fluorescence Competitive Binding Assay

Based on literature reports indicating that the elaiosomes of *S. tuberosa* and honeybee cuticular compounds share thirteen volatile semiochemicals, this study employed fluorescence competitive binding assays to determine the binding characteristics of VvelCSPs proteins to these thirteen volatiles. Fluorescence competitive binding assays were employed to characterize the interactions between VvelCSPs and 13 volatiles shared between the elaiosomes of *S. tuberosa* and the cuticular hydrocarbons of honeybees. These tests were performed in 1 cm path length quartz cuvettes with an excitation wavelength of 337 nm and emission wavelength scanning from 400–450 nm. The volatiles and the fluorescent probe 1-NPN (1-N-phenylnaphthylamine), listed in Supplementary Table S2, were dissolved in methanol to prepare 100 mM stock solutions, which were then diluted to 1 mM working solutions [25]. To remove any potential endogenous ligands, the purified proteins were delipidated by three washes with an equal volume of dichloromethane (1:1 *v*/*v*). Each wash involved a 10-min incubation, and the process was followed by evaporation of the residual solvent under a gentle stream of nitrogen. Purified CSPs were resuspended in 20 mM Tris-HCl buffer (pH 7.5) to a final concentration of 2 μM. Binding parameters for CSP-NPN were determined by titration: 2 mL of CSP solution (2 μM) was titrated with 1-NPN (final concentrations: 2−24 μM). Maximum fluorescence intensity was recorded, and the dissociation constant was calculated. For competitive binding assays, 2 mL of CSP solution (2 μM) was mixed with 2 μL of 1-NPN solution (final concentration: 1 μM), followed by stepwise addition of volatiles (final concentrations: 2−24 μM). After incubation in the dark for 2 min, fluorescence quenching was measured. Half-maximal inhibitory concentration (IC_50_) values for volatiles were calculated from the competition curves, and their binding constants (K_i_) were subsequently derived. The presented data, including IC_50_ and K_i_ values, originate from a single experiment under uniform conditions to ensure comparability (Supplementary Table S3).

### 2.6. Structural Modeling and Molecular Docking

The three-dimensional structures of VvelCSP1 and VvelCSP4 were modeled using the AlphaFold3 server (https://deepmind.google/science/alphafold/alphafold-server/, accessed on 24 March 2025) and validated via Ramachandran plots generated by the PROCHECK tool in sAVES 6.0 (https://www.playmolecule.com/deepsite/, accessed on 24 March 2025). Volatile compounds were downloaded from the ZINC database (https://zinc.docking.org/, accessed on 24 March 2025). The Deepsite server was used to predict and evaluate the potential ligand binding pockets of the simulated structure of the VvelCSPs. Docking was performed using AutoDock Vina (v1.5.6), with parameters configured based on the protein structure and the active site. Binding free energy (kcal/mol) was used as the evaluation index, and the optimal binding conformations were screened and saved [26]. The optimal binding poses were analyzed for hydrogen bonds and hydrophobic interactions using the PLIP server (https://plip-tool.biotec.tu-dresden.de/plip-web/plip/index, accessed on 26 March 2025) and PyMOL (v3.1.3.1).

## 3. Results

### 3.1. Sequence Conservation and Key Structural Motifs

**Figure 1:**
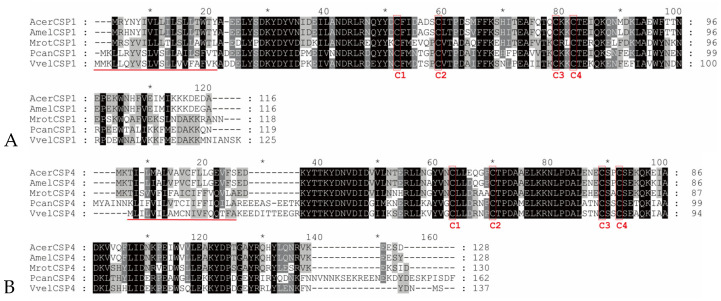
Alignment of amino acid sequence of VvelCSP1 (**A**) and VvelCSP4 (**B**) with homologous proteins from other hymenopteran insects. Identical amino acids are highlighted with a black background. Conserved cysteines are marked with red boxes. The signal peptides of VvelCSP1 and VvelCSP4 are indicated with red lines. Vvel, *Vespa velutina auraria*; Acer, *Apis cerana*; Amel, *Apis mellifera*; Mrot, *Megachile rotundata*; Pdun, *Polistes canadensis*.

**Figure 2:**
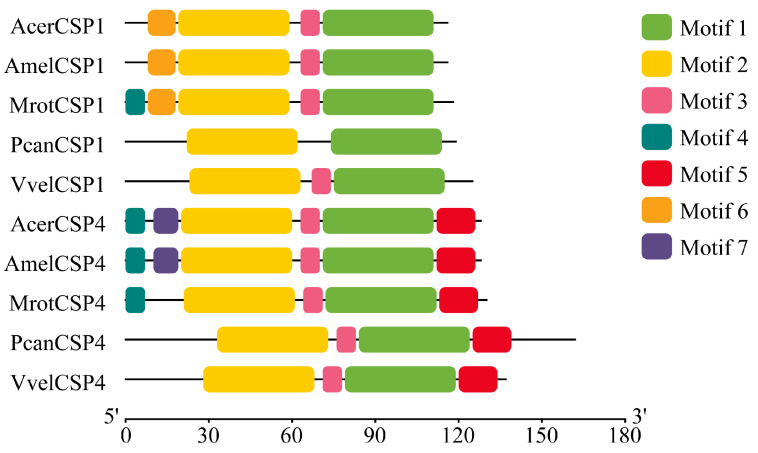
Conserved motif patterns of VvelCSP1 and VvelCSP4 with homologous proteins from other hymenopteran insects. Colored boxes represent conserved motifs.

Two full-length chemosensory protein genes, *VvelCSP1* and *VvelCSP4*, were cloned and identified from *V. velutina auraria*. Sequence analysis revealed that the full-length VvelCSP1 encodes a 125-amino acid peptide (14.75 kDa, pI 4.91), while the full-length VvelCSP4 encodes a 137-amino acid peptide (15.89 kDa, pI 4.77). Both precursor proteins contain an N-terminal signal peptide and possess the typical four-cysteine motif, suggesting their potential roles in olfactory recognition (Figure 1). Multiple sequence alignment with homologous proteins from other Hymenopteran insects showed that VvelCSP1 and VvelCSP4 exhibited high sequence identities of 67.52% and 60.86%, respectively. Furthermore, VvelCSP1 motifs follow a conserved “4-6-2-3-1” pattern, whereas VvelCSP4 motifs are arranged in a “4-7-2-3-1-5” pattern (where numbers are specific Motif IDs generated by MEME), with most core motifs in both proteins being highly conserved (Figure 2). This high degree of sequence similarity and motif conservation suggests a close phylogenetic relationship among these CSPs and implies a conserved function in olfactory recognition.

### 3.2. Expression and Purification of VvelCSPs

**Figure 3:**
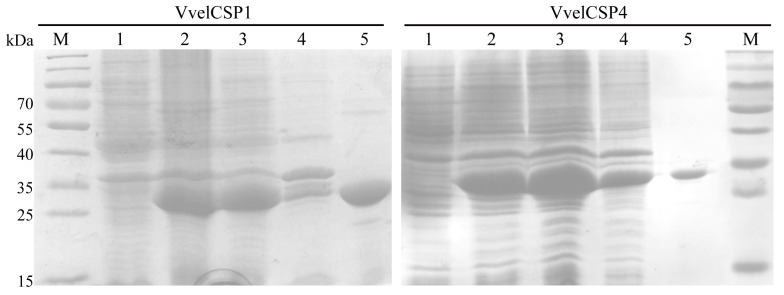
SDS-PAGE analysis of recombinant VvelCSP1 and VvelCSP4. Lane M, Protein Marker; Lane 1, Pre-induction; Lane 2, Post-induction; Lane 3, supernatant; Lane 4, pellet; Lane 5, Purified protein.

The VvelCSP1 and VvelCSP4 proteins were successfully expressed in the prokaryotic expression system, whose induction was carried out at 25 °C for 7 h with an IPTG concentration of 0.8 mM. Under these conditions, the target proteins were successfully expressed in the supernatant. The supernatant containing the target proteins was obtained through low-temperature high-pressure crushing and centrifugation techniques. Finally, the proteins expressed in the supernatant were purified by Ni-NTA affinity chromatography, and then the salt ions were removed by dialysis with a Tris-HCl solution to obtain the purified proteins. SDS-PAGE analysis confirmed the high purity of the recombinant VvelCSP1 and VvelCSP4. The proteins migrated as single bands at their expected molecular weights of approximately 32 kDa for VvelCSP1 and 33 kDa for VvelCSP4, confirming their successful expression and purification for subsequent experiments (Figure 3).

### 3.3. Fluorescence Competitive Binding Analysis of VvelCSPs

**Figure 4:**
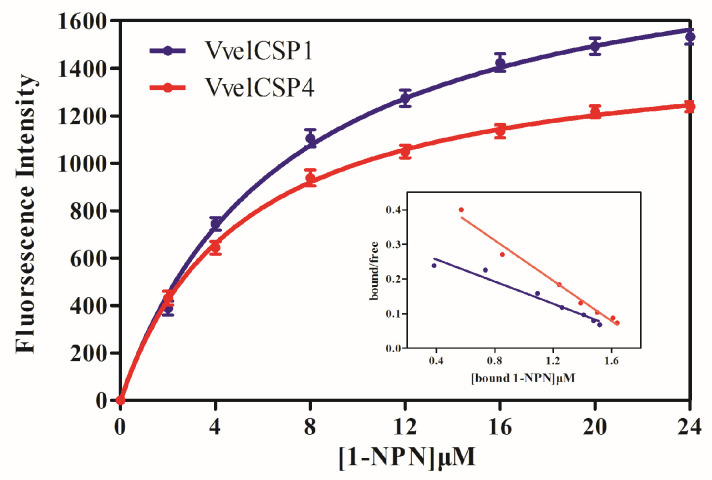
Binding curves and Scatchard plots of 1-NPN with VvelCSPs.

Fluorescence competitive binding experiments were employed to investigate the binding characteristics between the chemosensory proteins (VvelCSP1 and VvelCSP4) and the volatiles to further explore the olfactory molecular mechanism by which they recognize volatile compounds of volatile compounds from the elaiosomes of *S. tuberosa*. First, the binding curves of 1-NPN with the CSP1 and CSP4 proteins were determined (Figure 4). Analysis using the Scatchard equation revealed that the fluorescence values corresponding to the binding of CSPs to 1-NPN were singular, and the fluorescence values gradually tended towards saturation as the concentration increased. Therefore, 1-NPN could be used as a fluorescence probe in the fluorescence competitive binding experiments between the CSPs and the volatiles.

**Figure 5:**
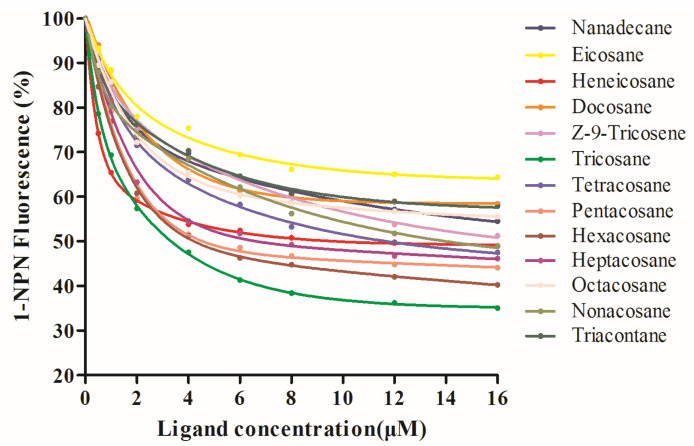
Competitive binding curves of VvelCSP1 to ligands.

**Figure 6:**
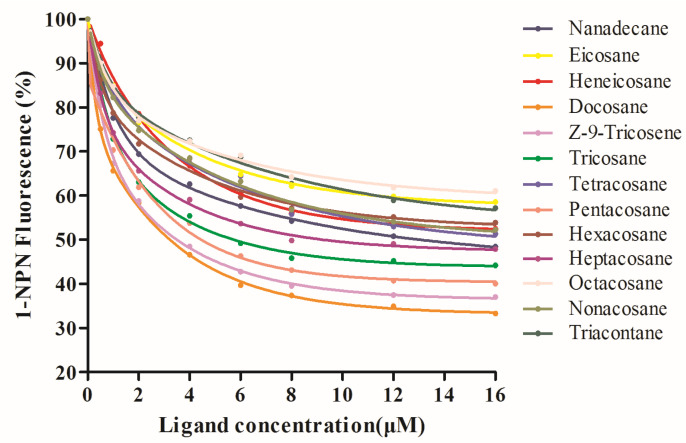
Competitive binding curves of VvelCSP4 to ligands.

The binding abilities of 13 volatiles with VvelCSP1 and VvelCSP4 proteins were measured (Figure 5 and Figure 6), and their IC_50_ values and dissociation constant K_i_ values were calculated using formulas, as shown in Table 1. Analysis results of the fluorescence competitive binding experiments with the 13 volatiles showed that VvelCSP1 and VvelCSP4 have significantly different binding abilities for various substrates, although both proteins showed a binding preference for volatile compounds from the elaiosomes of *S. tuberosa*. To facilitate the discussion of binding affinities, we adopted operational definitions where binding was considered strong if K_i_ < 5 μM, moderate for K_i_ values between 5 and 10 μM, and weak if K_i_ > 10 μM. According to these criteria, VvelCSP1 exhibits great binding abilities with tricosane (K_i_ = 3.07 μM), pentacosane (3.87 μM), hexacosane (3.68 μM), and heptacosane (4.40 μM), followed by moderate affinity for heneicosane and tetracosane, and weak binding to nanadecane, Z-9-Tricosene and nonacosane. In contrast, VvelCSP4 bound well to docosane, Z-9-tricosene, tricosane, pentacosane and heptacosane, with K_i_ values of 2.73 μM, 2.97 μM, 3.85 μM, 3.49 μM and 4.60 μM, respectively, while showing moderate affinity for nanadecane and weaker binding to the remaining compounds. These results indicate that VvelCSP1 and VvelCSP4 exhibit excellent binding capabilities with most volatile compounds from the elaiosomes of *S. tuberosa*. Notably, a striking ligand specificity was observed, with hexacosane binding strongly only to VvelCSP1 and docosane exclusively to VvelCSP4. This suggests that VvelCSP1 and VvelCSP4 function as the specific receptors for hexacosane and docosane, respectively.

### 3.4. Structural Modeling and Molecular Docking of VvelCSP1 and VvelCSP4 with Different Ligands

**Figure 7:**
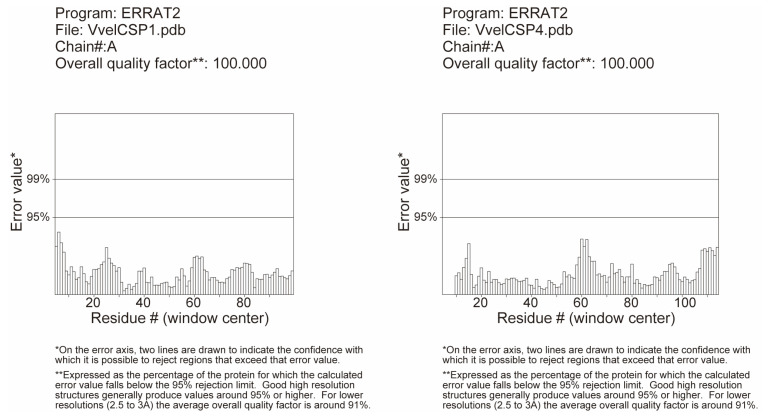
ERRAT results of the constructed 3D model of VvelCSP1 and VvelCSP4.

**Figure 8:**
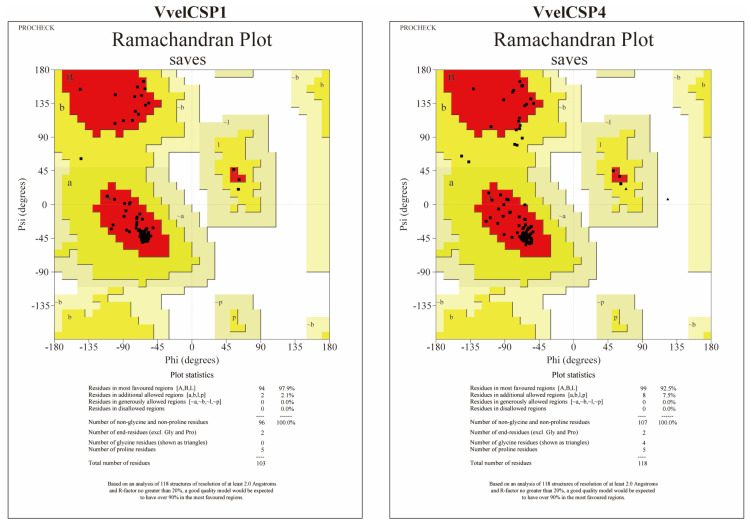
Ramanchandran plot of the constructed 3D model of VvelCSP1 and VvelCSP4.

The three-dimensional structures of VvelCSP1 and VvelCSP4 were successfully predicted using the AlphaFold3 algorithm. To ensure the reliability of the models, a rigorous quality assessment was conducted, beginning with the metrics intrinsic to AlphaFold. The primary validation, the predicted Local Distance Difference Test (pLDDT), yielded average scores of 88.95 for VvelCSP1 and 84.56 for VvelCSP4, indicating high confidence in the predicted structures. The few regions with lower pLDDT scores were observed to be confined to the flexible N- and/or C-termini. In addition, the models were cross-validated using classical stereochemical checks. ERRAT analysis showed that the overall quality factor for both models reached 100%. Moreover, PROCHECK Ramachandran plot analysis indicated that 97.9% and 92.5% of residues in VvelCSP1 and VvelCSP4, respectively, were located in the most favored regions, with no residues in disallowed regions (Figure 7 and Figure 8). Taken together, this comprehensive validation confirms that the constructed protein models possess high reliability and are suitable for subsequent molecular docking studies.

**Figure 9:**
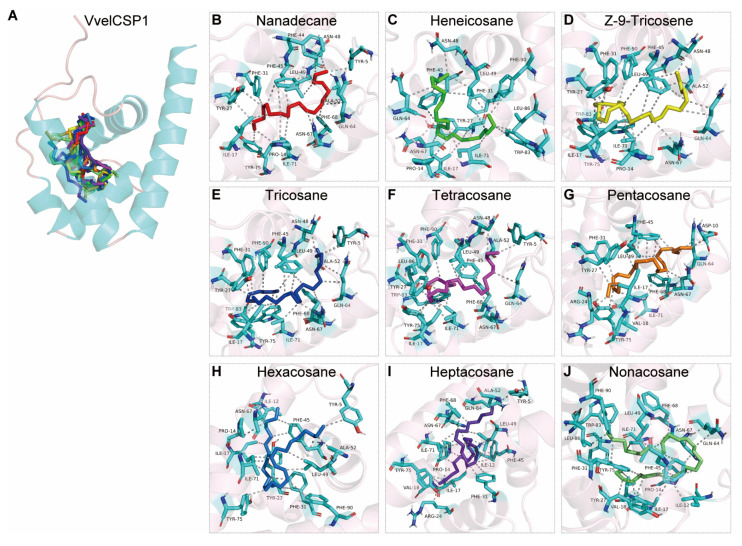
The binding mode of VvelCSP1 with bee cuticular volatile compounds. (**A**) The 3D structural model of VvelCSP1 indicating the interacting positions of ligands. (**B**–**J**) Illustrations show the amino acid residues of VvelCSP1 binding with nanadecane, heicosane, Z-9-tricosene, tricosane, tetracosane, pentacosane, hexacosane, heptacosane and nonacosane, respectively. The ligands are depicted using color-coded sticks. The hydrophobic interactions are marked with gray dotlines.

**Figure 10:**
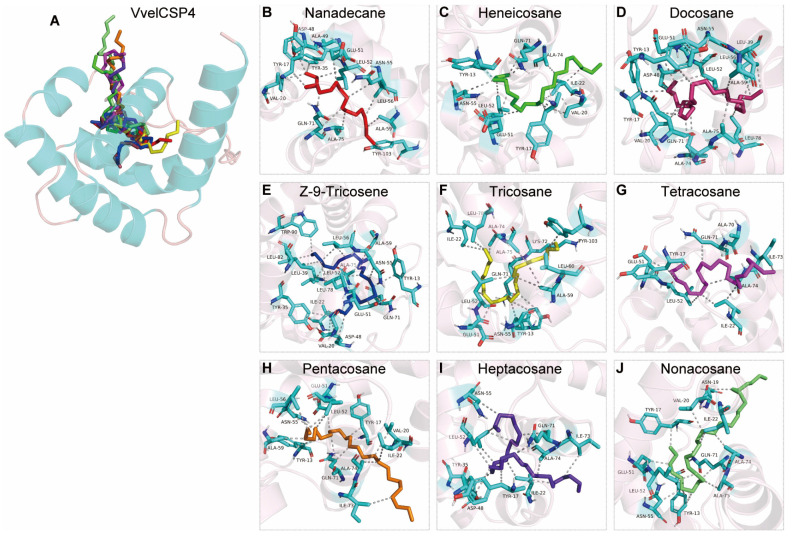
The binding mode of VvelCSP4 with bee cuticular volatile compounds. (**A**) The 3D structural model of VvelCSP4 indicating the interacting positions of ligands. (**B**–**J**) Illustrations show the amino acid residues of VvelCSP4 binding with nanadecane, heicosane, docosane, Z-9-tricosene, tricosane, tetracosane, pentacosane, heptacosane and nonacosane, respectively. The ligands are depicted using color-coded sticks. The hydrophobic interactions are marked with gray dotlines.

Molecular docking analysis revealed that the binding of VvelCSP1 and VvelCSP4 to honeybee cuticular volatiles is primarily driven by hydrophobic interactions. The predicted binding free energy spanned a wide range, from −8.22 to −3.70 kcal/mol, indicating varying degrees of binding affinity among the ligands (Table 2). The two proteins exhibited distinct differences in their ligand-binding sites. For VvelCSP1, the core binding pocket is composed of residues Ile17, Phe31, Phe45, Leu49, Asn67, and Ile71, which collectively participate in ligand recognition and binding (Figure 9 and Supplementary Table S4). Additionally, residues such as Pro14, Tyr27, Ala52, Gln64, Phe68, Tyr75 and Phe90 also play a key role in stabilizing the binding with most ligands (Figure 9). In contrast, for VvelCSP4, Leu52 and Gln71 displayed broad ligand recognition capabilities, interacting with all nine volatile compounds. Meanwhile, residues including Tyr13, Tyr17, Val20, Ile22, Glu51, Asn55, and Ala74 formed an extensive hydrophobic network, significantly enhancing the stability of the protein–ligand complexes, which corresponds to the more favorable (lower) binding energy values (Figure 10 and Supplementary Table S4).

## 4. Discussion

Social insects rely on olfactory and visual cues to locate and evaluate potential nesting sites, mates and resources; for example, the Japanese giant hornet (*Vespa mandarinia japonica*) utilizes volatile odor molecules to detect and locate prey through olfaction [27,28]. Hymenopteran insects possess a relatively complex and refined pheromone-based chemical communication system, wherein olfactory protein families such as CSPs, OBPs and ORs play crucial roles in the chemosensory perception of environmental volatile cues [29,30,31]. In this study, we investigated the molecular basis of olfactory recognition of honeybee cuticular volatiles and *S. tuberosa* seed appendage volatiles in the hornet through functional analysis of two CSP genes. Sequence analysis of *V. velutina auraria* CSPs revealed that the predicted amino acid sequences contain four conserved cysteine residues, a characteristic feature of insect chemosensory proteins. Furthermore, VvelCSP1 and VvelCSP4 were found to contain two and one tryptophan residues, respectively. These residues play a crucial role in fluorescence quenching assays for evaluating ligand binding, as previously documented in CSP16 of *Bombyx mori* [32], CSP1–3 of *A. mellifera* [33], CSP3 of *Microplitis mediator* [34], CSP3 of *Cotesia ruficrus* [35] and CSP1 of *Reticulitermes aculabialis* [36]. Homology alignment demonstrated that VvelCSP1 and VvelCSP4 share high sequence identity with their orthologs in other hymenopteran insects, indicating close evolutionary relationships and further supporting the high conservation of insect CSPs during evolution [33].

Ligand-binding studies on chemosensory proteins (CSPs) in hymenopteran insects remain limited, with particularly few reports on CSPs in vespid wasps and many olfactory functions still uncharacterized. Previous binding assays have shown that *Polistes dominula* CSPs exhibit strong specificity and sensitivity toward amines and alcohols with 12–18 carbon chains [37], while CSPs in *M. mediator* (MmedCSPs) can bind various plant volatiles and insect pheromones [34]. Chen et al. revealed that hornets mediate seed dispersal of the medicinal plant *S. tuberosa*, and that the elaiosomes of *S. tuberosa* share thirteen identical volatile compounds with honeybee cuticular surfaces [24,38]. Since hornets primarily feed on the elaiosomes of *S. tuberosa* seeds, we selected these 13 specific volatiles from honeybee body surfaces and the elaiosomes of *S. tuberosa* seeds for competitive fluorescence binding assays with VvelCSP1 and VvelCSP4. Our results demonstrate that VvelCSP1 and VvelCSP4 exhibit varying binding affinities to all 13 volatiles, indicating that these CSPs are involved in recognizing and locating elaiosome-derived odor cues. We propose that *S. tuberosa* seeds may mimic the odor profiles of honeybee cuticles to attract hornets, thereby facilitating seed predation and potentially long-distance dispersal. This hypothesis is supported by earlier findings that *Dendrobium sinense* releases Z-11-eicosen-1-ol, a component of honeybee alarm pheromone, to attract the hornet *V. bicolor* for pollination [39]. Similarly, the elaiosomes of *S. tuberosa* contain analogous compounds that attract hornets, supporting the idea that “smelling like prey” could be an evolved strategy used by plants to manipulate hornet behavior for seed dispersal.

During long-term evolution, CSPs have developed adaptive divergence across insect species, with accumulating evidence indicating that amino acid substitutions within their binding pockets serve as a primary driver of such functional variation [35,40]. To elucidate the molecular mechanisms underlying ligand recognition and binding by these chemosensory proteins, we performed homology modeling and molecular docking analyses for two CSPs in *V. velutina auraria*. The modeled structures of VvelCSPs adopt a canonical fold consisting of five or six α-helices, exhibiting overall conservation with the tertiary structures of CSPs from *A*. *cerana*, as previously reported [40,41]. Molecular docking results revealed binding affinities generally consistent with data from fluorescence competitive binding assays. However, a direct quantitative correlation between the calculated binding energies and the experimental K_i_ values was not observed, as exemplified by the similar K_i_ values but disparate docking scores of docosane and hexacosane. This is a known limitation of docking scoring functions, which are more adept at predicting binding modes than precisely ranking ligand affinities. The dissociation constants (K_i_) determined in this study for VvelCSP1 and VvelCSP4 with various alkanes ranged from 2.73 μM to 14.74 μM. This affinity range is physiologically relevant and consistent with reported values for CSPs binding hydrocarbons in other insects; for example, CsasCSP16 from *Carposina sasakii* binds pentadecane with a K_i_ of 3.5 μM [42]. These data support the conserved role of CSPs as transporters of hydrophobic semiochemicals and suggest that VvelCSP1 and VvelCSP4 efficiently transport specific cuticular hydrocarbons in *V. velutina auraria*.

Importantly, the docking models are highly effective at explaining the structural basis for these binding patterns. The structure–activity relationship is governed by the structural match between the ligand and the binding pocket. VvelCSP4 exhibits clear size selectivity, peaking at C22-C23, whereas VvelCSP1 shows a more complex profile, where C26 binds weakly due to an unfavorable conformation. The lack of binding in some alkanes is a direct result of severe structural mismatch: ligands that are too short fail to make stable contacts, those that are too long are sterically hindered, and certain intermediate-length ligands cannot adopt a stable conformation. Thus, both binding affinity and the ability to bind are determined by the degree of ligand-pocket complementarity. Specifically, hydrophobic interactions predominantly mediated the binding between VvelCSPs and alkane-type volatiles. Notably, nine volatiles consistently interacted with Ile17, Phe31, Phe45, Leu49, Asn67 and Ile71 in VvelCSP1, while for VvelCSP4, the interacting residues were Leu52 and Gln71, suggesting that VvelCSP1 and VvelCSP4 may contribute to seed recognition and dispersal behavior in *V. velutina auraria*. However, in vivo functional validation through behavioral assays or electrophysiological recordings will be necessary to confirm their physiological roles. Further validation through site-directed mutagenesis coupled with fluorescence binding assays will be essential to confirm the functional significance of these putative binding sites.

## 5. Conclusions

In summary, our findings demonstrate that VvelCSP1 and VvelCSP4 play pivotal roles in the seed recognition and dispersal behavior of *V. velutina auraria* toward *S. tuberosa* seeds. These results provide mechanistic insight into the molecular regulation of olfactory-mediated physiological behaviors, such as prey hunting and seed dissemination in this hornet species. Elucidating the function of these chemosensory proteins advances our understanding of chemical communication pathways in predatory hymenopterans and offers a scientific foundation for deciphering pheromone transmission and perception mechanisms. Furthermore, this study establishes a basis for developing novel attractants or repellents targeting hornet behavior, with potential applications in ecological management and species-specific control strategies.

## Figures and Tables

**Table 1:** Binding constants of VvelCSP1 and VvelCSP4 with ligands.

No.	Compounds	VvelCSP1		VvelCSP4	
		IC_50_ (μM)	K_i_ (μM)	IC_50_ (μM)	K_i_ (μM)
1	Nanadecane	18.23	14.53	10.44	7.89
2	Eicosane	-	-	-	-
3	Heneicosane	11.27	9.02	19.76	14.74
4	Docosane	-	-	3.68	2.73
5	Z-9-Tricosene	17.35	13.85	3.97	2.97
6	Tricosane	3.85	3.07	5.09	3.85
7	Tetracosane	11.23	8.95	18.93	14.25
8	Pentacosane	4.83	3.87	4.65	3.49
9	Hexacosane	4.63	3.68	-	-
10	Heptacosane	5.53	4.40	6.20	4.60
11	Octacosane	-	-	-	-
12	Nonacosane	14.56	11.63	20.37	15.07
13	Triacontane	-	-	-	-

IC_50_, ligand concentration displacing 50% of the fluorescence intensity of the CSPs/NPN complex; K_i_, dissociation constant; “-“ means the protein did not bind with the ligands in the assay.

**Table 2:** Docking results for VvelCSP1 and VvelCSP4 with ligands.

Compounds	VvelCSP1	VvelCSP4
	Binding Energy (kcal/mol)	Binding Energy (kcal/mol)
Nanadecane	−5.86	−7.14
Heneicosane	−5.71	−7.49
Docosane	-	−8.22
Z-9-Tricosene	−5.85	−8.28
Tricosane	−6.41	−7.72
Tetracosane	−6.1	−4.74
Pentacosane	−4.32	−5.14
Hexacosane	−4.18	-
Heptacosane	−6.35	−4.00
Nonacosane	−3.70	−5.00

## Data Availability

The original contributions presented in this study are included in the article/Supplementary Material. Further inquiries can be directed to the corresponding author.

## Supplementary Materials

The following supporting information can be downloaded at: https://www.mdpi.com/article/10.3390/genes16111265/s1, Supplementary Table S1: Primer sequences of VvelCSP1 and VvelCSP4; Table S2: All chemicals lists as well as the detailed information used in this study; Supplementary Table S3: Raw data of the fluorescent competitive binding assay; Supplementary Table S4: Docking results for VvelCSP1 and VvelCSP4 with ligands.
