# Supplementary material for: The Chemoreceptive Molecular Mechanism Underlying CSP-Mediated Recognition of Seed Elaiosome from Stemona tuberosa by Hornets
Source: Genes (Basel). 2025 Oct 27;16(11):1265. doi: 10.3390/genes16111265 (PMC12652730; doi:10.3390/genes16111265)
Supplement: Supplementary file 1 [file genes-16-01265-s001.zip › genes-3930640-supplementary.pdf]

Supplementary Table S1 Primer sequences of VvelCSP1 and VvelCSP4

Genes	Forward primer (5′-3′)	Reverse primer (5′-3′)
VvelCSP1	CGCGGATCCATGATGAAGTTACTCCA ATATGTCT	CCGCTCGAGTTTCGAATTTGCTATGTTC ATT
VvelCSP4	CGCGGATCCATGTTAATCCTTGTTAT ATTGGC	CCGAAGCTTGGACATATTATCATAGTT GAATTTA

Supplementary Table S2 All chemicals lists as well as the detailed information used in this study

Chemicals	Company	CAS Number	Purity
1-NPN	TCI	90-30-2	>98%
IPTG	Sigma	367-93-1	≥99%
Nanadecane	TCI	629-92-5	>98.0%
Eicosane	TCI	112-95-8	>98%
Heneicosane	TCI	629-94-7	>99.5%
Docosane	TCI	629-97-0	>99.0%
Z-9-Tricosene	Sigma	27519-02-4	>97%
Tricosane	TCI	638-67-5	>99.0%
Tetracosane	TCI	646-31-1	>99.0%
Pentacosane	TCI	629-99-2	>97.0%
Hexacosane	TCI	630-01-3	>99.0%
Heptacosane	TCI	593-49-7	>97.0%
Octacosane	TCI	630-02-4	>98.0%
Nonacosane	TCI	630-03-5	>98.0%
Triacontane	TCI	638-68-6	>98.0%
α-terpineol	TCI	98-55-5	>95%

Supplementary Table S3 Raw data of the fluorescent competitive binding assay

Chemosensory protein	Chemicals	The Concentration of Chemicals (μM)								
		0	0.5	1	2	4	6	8	12	16
VvelCSP1	Nanadecane	245.70	223.11	209.38	178.27	171.35	158.50	148.00	140.37	133.91
	Eicosane	264.60	246.85	234.09	206.27	199.36	183.72	175.07	172.10	170.22
	Heneicosane	257.30	190.87	168.28	152.31	138.38	134.83	130.66	127.58	125.86
	Docosane	262.40	246.77	231.89	197.76	171.56	161.14	158.53	154.82	153.17
	Z-9-Tricosene	234.70	214.80	200.54	176.56	164.98	151.46	137.73	126.20	120.19
	Tricosane	245.50	193.01	170.16	140.89	116.63	101.34	94.22	88.89	86.00
	Tetracosane	247.30	225.09	200.88	176.79	157.66	144.05	131.64	122.98	117.42
	Pentacosane	278.30	242.52	219.99	165.83	143.30	135.17	129.88	124.70	122.60
	Hexacosane	223.50	189.29	171.76	135.79	114.55	103.35	99.94	93.92	89.96
	Heptacosane	251.40	222.51	205.55	159.00	136.99	130.11	123.81	117.37	115.92
	Octacosane	224.40	200.77	192.40	162.06	146.64	135.22	131.33	127.20	124.62
	Nonacosane	245.60	215.85	197.03	181.17	168.33	152.52	138.03	127.11	120.08
	Triacontane	236.90	211.63	196.09	180.51	166.41	152.93	143.85	139.39	137.14
VvelCSP4	Nanadecane	267.20	238.31	207.32	185.38	167.28	153.91	144.81	135.70	129.34
	Eicosane	214.60	192.14	182.38	164.23	154.96	139.00	133.35	128.24	125.62
	Heneicosane	267.30	252.45	226.44	209.89	178.34	161.57	149.85	143.60	140.19
	Docosane	274.30	205.93	179.89	159.86	127.80	108.85	102.51	95.75	91.32
	Z-9-Tricosene	226.30	182.16	152.13	132.92	109.71	96.72	89.44	84.83	83.78
	Tricosane	243.70	207.46	177.45	153.67	134.98	119.90	111.62	110.10	107.68
	Tetracosane	238.40	216.50	198.86	178.28	160.59	153.54	132.96	126.27	122.27
	Pentacosane	259.30	211.45	182.28	160.44	139.39	119.91	111.74	105.45	103.82
	Hexacosane	247.30	213.35	194.71	177.42	169.19	147.61	141.30	136.25	133.13
	Heptacosane	262.30	218.17	194.71	172.10	154.91	140.68	130.60	128.65	125.52
	Octacosane	252.40	227.35	213.99	194.19	182.58	174.55	162.14	156.02	154.11
	Nonacosane	239.60	212.97	196.97	179.08	164.16	151.46	136.04	129.53	125.16
	Triacontane	236.50	216.07	200.62	183.85	171.56	162.42	148.29	139.28	135.24

Supplementary Table S4 Docking results for VvelCSP1 and VvelCSP4 with ligands

Chemosensory protein	Ligand	Interacting residues
VvelCSP1	Nanadecane	Tyr5, Pro14, Ile17, Tyr27, Phe31, Phe44, Phe45, Asn48, Leu49, Ala52, Gln64, Asn67, Phe68, Ile71, Tyr75
	Heneicosane	Pro14, Ile17, Tyr27, Phe31, Phe45, Asn48, Leu49, Gln64, Asn67, Ile71, Trp83, Leu86, Phe90
	Z-9-Tricosene	Pro14, Ile17, Tyr27, Phe31, Phe45, Asn48, Leu49, Ala52, Gln64, Asn67, Ile71, Tyr75, Trp83, Phe90
	Tricosane	Tyr5, Ile17, Tyr27, Phe31, Phe45, Asn48, Leu49, Ala52, Gln64, Asn67, Phe68, Ile71, Tyr75, Trp83, Phe90
	Tetracosane	Tyr5, Ile17, Tyr27, Phe31, Phe45, Asn48, Leu49, Ala52, Gln64, Asn67, Phe68, Ile71, Tyr75, Trp83, Leu86, Phe90
	Pentacosane	Asp10, Ile17, Val18, Tyr27, Phe31, Phe45, Leu49, Gln64, Asn67, Phe68, Ile71, Tyr75
	Hexacosane	Tyr5, Ile12, Pro14, Ile17, Tyr27, Phe31, Phe45, Leu49, Ala52, Asn67, Ile71, Tyr75, Phe90
	Heptacosane	Tyr5, Ile12, Pro14, Ile17, Val18, Phe31, Phe45, Leu49, Ala52, Gln64, Asn67, Phe68, Ile71, Tyr75
	Nonacosane	Ile12, Pro14, Ile17, Val18, Tyr27, Phe31, Phe45, Leu49, Gln64, Asn67, Phe68, Ile71, Tyr75, Trp83, Leu86, Phe90
VvelCSP4	Nanadecane	Tyr17, Val20, Tyr35, Asp48, Glu51, Leu52, Asn55, Leu56, Ala59, Gln71, Ala75, Tyr103
	Heneicosane	Tyr13, Tyr17, Val20, Ile22, Glu51, Leu52, Asn55, Gln71, Ala74
	Docosane	Tyr13, Tyr17, Val20, Ile22, Leu39, Asp48, Glu51, Leu52, Asn55, Leu56, Ala59, Gln71, Ala74, Ala75, Ala78
	Z-9-Tricosene	Tyr13, Ile22, Glu51, Leu52, Asn55, Ala59, Leu60, Gln71, Lys72, Ala74, Ala75, Ala78, Tyr103
	Tricosane	Tyr13, Val20, Ile22, Tyr35, Leu39, Asp48, Glu51, Leu52, Asn55, Leu56, Ala59, Gln71, Ala75, Ala78, Leu82, Trp90
	Tetracosane	Tyr17, Ile22, Glu51, Leu52, Ala70, Gln71, Ile73, Ala74
	Pentacosane	Tyr13, Tyr17, Val20, Ile22, Glu51, Leu52, Asn55, Leu56, Ala59, Gln71, Ile73, Ala74
	Heptacosane	Tyr17, Ile22, Tyr35, Asp48, Leu52, Asn55, Gln71, Ile73, Ala74
	Nonacosane	Tyr13, Tyr17, Asn19, Val20, Ile22, Glu51, Leu52, Asn55, Gln71, Ala74
